# Maternal Clinical Diagnoses and Hospital Variation in the Risk of Cesarean Delivery: Analyses of a National US Hospital Discharge Database

**DOI:** 10.1371/journal.pmed.1001745

**Published:** 2014-10-21

**Authors:** Katy B. Kozhimannil, Mariana C. Arcaya, S. V. Subramanian

**Affiliations:** 1 Division of Health Policy and Management, University of Minnesota School of Public Health, Minneapolis, Minnesota, United States of America; 2 Department of Social and Behavioral Sciences, Harvard School of Public Health, Boston, Massachusetts, United States of America; Cambridge University, United Kingdom

## Abstract

**Background:**

Cesarean delivery is the most common inpatient surgery in the United States, where 1.3 million cesarean sections occur annually, and rates vary widely by hospital. Identifying sources of variation in cesarean use is crucial to improving the consistency and quality of obstetric care. We used hospital discharge records to examine the extent to which variability in the likelihood of cesarean section across US hospitals was attributable to individual women's clinical diagnoses.

**Methods and Findings:**

Using data from the 2009 and 2010 Nationwide Inpatient Sample from the Healthcare Cost and Utilization Project—a 20% sample of US hospitals—we analyzed data for 1,475,457 births in 1,373 hospitals. We fitted multilevel logistic regression models (patients nested in hospitals). The outcome was cesarean (versus vaginal) delivery. Covariates included diagnosis of diabetes in pregnancy, hypertension in pregnancy, hemorrhage during pregnancy or placental complications, fetal distress, and fetal disproportion or obstructed labor; maternal age, race/ethnicity, and insurance status; and hospital size and location/teaching status.

The cesarean section prevalence was 22.0% (95% confidence interval 22.0% to 22.1%) among women with no prior cesareans. In unadjusted models, the between-hospital variation in the individual risk of primary cesarean section was 0.14 (95% credible interval 0.12 to 0.15). The difference in the probability of having a cesarean delivery between hospitals was 25 percentage points. Hospital variability did not decrease after adjusting for patient diagnoses, socio-demographics, and hospital characteristics (0.16 [95% credible interval 0.14 to 0.18]). A limitation is that these data, while nationally representative, did not contain information on parity or gestational age.

**Conclusions:**

Variability across hospitals in the individual risk of cesarean section is not decreased by accounting for differences in maternal diagnoses. These findings highlight the need for more comprehensive or linked data including parity and gestational age as well as examination of other factors—such as hospital policies, practices, and culture—in determining cesarean section use.

*Please see later in the article for the Editors' Summary*

## Abstract 2

Katy Kozhimannil and colleagues use a national database to examine the extent to which variability in cesarean section rates across the US from 2009–2010 was attributable to individual women's clinical diagnoses.

*Please see later in the article for the Editors' Summary*

## Editors' Summary

**Background:**

In an ideal world, all babies would be delivered safely and naturally through their mother's vagina. However, increasing numbers of babies are being delivered by cesarean section, a surgical operation in which the baby is delivered through a cut made in the mother's abdomen and womb. In the US, a third of all babies (about 1.3 million babies in 2011) are delivered this way. A cesarean section is usually performed when a vaginal birth would endanger the life of the mother or her unborn child because, for example, the baby is in the wrong position or the labor is not progressing normally. Some cesarean sections are performed as emergency procedures, but others are planned in advance when the need for the operation becomes clear during pregnancy. Although cesarean sections can save lives, women who deliver this way have higher rates of infection, pain, and complications in future pregnancies than women who deliver vaginally, and their babies can have breathing problems.

**Why Was This Study Done?:**

Currently, cesarean section rates vary widely from country to country and from hospital to hospital within countries. Careful assessment of the risks and benefits of cesarean delivery in individual patients can help to ensure that cesarean sections are used only when necessary, but changes to clinical and policy guidelines are also needed to ensure that cesarean delivery is neither overused nor underused. To guide these changes, we need to know whether cesarean section rates vary among hospitals because of case-mix differences (some hospitals may have high rates because they admit many women with complicated pregnancies, for example) or because of differences in modifiable nonclinical factors such as hospital policies and practices. In this retrospective multilevel analysis, the researchers examine whether the current wide variation in cesarean section rates across US hospitals is attributable to differences in maternal clinical diagnoses and patient characteristics or to hospital-level differences in the use of cesarean delivery.

**What Did the Researchers Do and Find?:**

For their study, the researchers used hospital discharge data on nearly 1.5 million births in 1,373 hospitals collected by the 2009 and 2010 US Nationwide Inpatient Sample database, which captures administrative data (for example, length of stay in hospital and clinical complications) from a representative sample of 20% of US hospitals. To assess the chances of cesarean delivery based on hospital and patient characteristics, researchers fitted these data to multilevel logistic regression statistical models. Among women with no prior cesarean deliveries, the (primary) cesarean section rate was 22%, whereas among the whole study population, it was 33% (women who have one cesarean delivery often have a cesarean section for subsequent deliveries). In unadjusted models that compared cesarean section rates between hospitals without considering patient characteristics, the between-hospital variance for primary cesarean section rate was 0.14. Put another way, the likelihood of an individual having a first cesarean delivery varied between 11% and 36% across the hospitals considered. After adjustment for maternal clinical diagnoses, maternal age and other socio-demographic factors, and hospital characteristics such as size, the between-hospital variance for the primary cesarean section rate was 0.16.

**What Do These Findings Mean?:**

The finding that the between-hospital variance for primary cesarean section rate did not decrease after adjusting for maternal characteristics (and other findings presented by the researchers) suggests that differences in case mix or pregnancy complexity may not drive the wide variability in cesarean section rates across US hospitals. However, the lack of information in the US Nationwide Inpatient Sample database on parity (the number of babies a woman has had) or gestational age (the length of time the baby has spent developing inside its mother) limits the strength of this conclusion. Both parity and gestational age strongly predict a woman's risk of a cesarean delivery. Thus, unmeasured differences in the parity of women admitted to different hospitals and/or the gestational age of their babies may be driving some of the variability in cesarean section rates across US hospitals. The lack of hospital-level information on obstetric care policies in the database also means that the many possible administrative explanations for variations across hospitals cannot be assessed. These findings therefore highlight the need for more comprehensive patient data to be collected (including information on parity and gestational age) and on hospital policies, practices, and culture before the variation in cesarean section rate across US hospitals can be fully understood and the use of cesarean delivery can be optimized.

**Additional Information:**

Please access these websites via the online version of this summary at http://dx.doi.org/10.1371/journal.pmed.1001745.

- This study is further discussed in a *PLOS Medicine* Perspective by Gordon C. S. Smith
- The American College of Obstetricians and Gynecologists provides a fact sheet for patients on cesarean birth
- The American College of Nurse-Midwives provides a fact sheet for pregnant women on preventing cesarean birth
- The US-based Childbirth Connection Project of the non-profit National Partnership for Women and Families has a booklet called “What Every Woman Should Know about Cesarean Section”
- The US-based non-profit Nemours Foundation provides detailed information about cesarean sections (in English and Spanish)
- The UK National Health Service Choices website provides information for patients about delivery by cesarean section
- MedlinePlus provides links to additional resources about cesarean section (in English and Spanish)
- The UK non-profit organization Healthtalkonline provides personal stories about women's experiences of cesarean delivery
- Information about the US Nationwide Inpatient Sample database is available

## Introduction

Cesarean delivery is the most common inpatient surgery in the United States [1], with rates having increased from 20.7% in 1996 to 32.9% in 2009 and stabilizing thereafter [2],[3]. Approximately 1.3 million American women had a cesarean delivery in 2011 [4]. Physicians commonly perform a cesarean delivery to avoid potential adverse events for women and infants, but the procedure also entails additional risks compared with vaginal delivery [5]. Women who deliver via cesarean section have higher rates of infection, pain, rehospitalization, breastfeeding challenges, and complications in future pregnancies [6]–[9]. Infants born by cesarean section have higher rates of hospital admission, need for ventilation, and respiratory morbidity [10]–[12]. The risks and benefits of cesarean delivery depend on clinical conditions that may be in flux, and assessing these risks and benefits requires careful attention to the individual needs of patients. However, there is broad consensus that improving the use of cesarean sections requires policy attention and clinical action [13]–[17]. In addition to individual-level differences in clinical needs, there are hospital-level differences in the use of cesarean sections, highlighted by the wide variations in cesarean section rates across hospitals [18]–[22].

A better understanding of the variation in procedure use can help to improve consistency, quality, and value in health care for the nearly 4 million US women and infants who receive childbirth care each year [23],[24]. Cesarean section use may vary across hospitals owing to case-mix differences, but nonclinical factors can also affect use, presenting opportunities to reduce medically unnecessary cesarean sections. Obstetricians and other maternity care providers recognize the urgent need to address both the rising rates and variability in the likelihood of cesarean delivery [13]–[15].

However, clinical and policy action in the US is limited by a lack of national evidence on whether variability is primarily driven by differences in case mix between hospitals; this lack of evidence is due, in part, to weaknesses in the population-based data infrastructure that do not reliably allow for linkages between birth registry data (containing important patient clinical information such as gestational age and parity) and hospital administrative data (containing hospital-level data on procedure use and individual procedures and diagnoses). This analysis makes use of hospital administrative data that contain information on diagnoses and hospital care, but data on characteristics such as parity and gestational age were not collected. Based on prior research on this topic in international settings and the increasing use of guidelines for the clinical management of women in labor, we hypothesized that we would uncover variability in the likelihood of a woman having a cesarean section across hospitals, and that this variability would be partially explained by the diagnoses of clinical conditions in individuals [22]. This analysis examined the extent to which variability in the risk of cesarean section across US hospitals was attributable to maternal clinical diagnoses.

## Methods

We used hospital discharge data in a retrospective multilevel analysis of individual risk of cesarean section across hospitals.

### Ethics Statement

Data for this analysis were de-identified, and as such, the study was granted exemption from review by the University of Minnesota Institutional Review Board (study number 1011E92980).

### Data and Study Population

We used data from the 2009 and 2010 Nationwide Inpatient Sample (NIS) from the Healthcare Cost and Utilization Project (HCUP) by the Agency for Healthcare Research and Quality (AHRQ). The NIS is an all-payer inpatient claims database designed to approximate a 20% stratified sample of US hospitals [25]. While it contains only administrative data, not clinical information, it is one of the most comprehensive national sources of information on hospital-based care in the US and has been regularly used in health services research [26]–[28]. The NIS is designed to approximate a 20% sample of all US community hospitals (non-federal, short-term, general, and other specialty hospitals, including obstetrics-gynecology, ear-nose-throat, orthopedic, and pediatric institutions). The sample includes both public hospitals and academic medical centers, but excludes short-term rehabilitation hospitals, long-term non-acute care hospitals, psychiatric hospitals, and alcoholism/chemical dependency treatment facilities. The hospitals in the NIS are identified using five strata (ownership/control, bed size categories defined by AHRQ, teaching status, urban/rural location, and US region) with sampling probabilities proportional to the number of US community hospitals in each stratum. HCUP provides weights that account for survey features to ensure national representativeness. Detailed information on the NIS dataset, methodology, and variables is publicly available (http://www.hcup-us.ahrq.gov/databases.jsp).

Our analyses focused on hospitals that reported discharges with neonatal and/or maternal diagnoses and procedures. From these hospitals, we used a validated methodology to identify hospital discharge records for obstetric deliveries [29]. Our final dataset included 1,475,457 births in 1,373 hospitals in 46 states, including 1,241,255 births to mothers with no prior cesarean sections.

### Variable Measurement

We calculated prevalence of cesarean delivery among two groups of women: (1) all women and (2) all women with no prior cesarean deliveries. We identified cesarean delivery using International Classification of Diseases, 9th revision (ICD-9) procedure codes (740X, 741X, 742X, 744X, 7499) as well as Diagnosis Related Group payment codes (370, 371), consistent with validated methods and prior research using the HCUP NIS data [29],[30]. We identified prior cesarean section by ICD-9 codes (65420, 65421, 65423). We calculated the individual likelihood of cesarean at each hospital as the percentage of cesarean sections among deliveries by all women and as the percentage of cesarean sections among deliveries by women with no prior cesarean sections (primary cesarean section) during 2009 and 2010. We also identified, as closely as these data allow, two other groups of women based on their risk status, consistent with AHRQ Inpatient Quality Indicator #33 [31] and used or identified in prior research [17],[19],[32]. These groups are (1) lower risk women, excluding those with preterm delivery (prior to 37 wk gestation; ICD-9 codes 6442, 64420, 64421), multiple gestation (ICD-9 codes 651, 6510X, 6511X, 6512X, 6513X, 6514X, 6515X, 6516X, 6518X, 6519X), fetal malpresentation (ICD-9 codes 652X, 6600X), and prior cesarean delivery, and (2) higher risk women, including those with preterm delivery, multiple gestation, fetal malpresentation, or prior cesarean section.

We used a unique hospital identification code to group deliveries by hospital. We also used hospital-specific data on bed size (as defined by AHRQ, accounting for geographic location), teaching status, and rural versus urban location. Hospital teaching status was based on information from the American Hospital Association's Annual Survey of Hospitals. Classification of hospitals as either urban or rural was based on Core Based Statistical Area codes from 2000 census data. Measurement of hospital characteristics replicated previously published studies using HCUP data [19],[27]–[30], and detailed information on each of these data elements is available on the HCUP website (http://www.hcup-us.ahrq.gov/databases.jsp). We also included fixed effects for state in fully adjusted models.

Individual-level covariates are based on administrative records, ICD-9 diagnosis and procedure codes, and Clinical Classifications Software (CCS) codes, developed by HCUP for use with ICD-9 codes. Covariates include maternal age, race/ethnicity, and insurance status (primary payer: private insurance, Medicare, Medicaid, self-pay/uninsured, or other), and maternal and infant medical conditions, including diagnoses of the following complications of pregnancy, labor, and delivery: diabetes in pregnancy (both diabetes mellitus and gestational diabetes; ICD-9 codes 6488XX, 250XX), hypertension in pregnancy (including pre-eclampsia and eclampsia; ICD-9 codes 6420X, 6421X, 6422X, 6423X, 6424X, 6425X, 6426X, 6424, 6425, 6426, 6426XX), hemorrhage during pregnancy or placental complications (including placenta previa and placenta accreta; CCS code 182), fetal disproportion or obstruction of labor (CCS code 188), and fetal distress (CCS code 190). Race/ethnicity is self-reported; specific response categories vary by state but are harmonized by HCUP into the following mutually exclusive categories: black, white, Hispanic, Asian, Native American, and other [25],[26]. In this study, race/ethnicity is included as a factor connected to cultural preferences and practices regarding childbirth.

### Analysis

Hospital cesarean section rates for each of the four groups described above (all women, all women with no prior cesarean, lower risk women, and higher risk women) were graphed using funnel plots, by the number of annual deliveries in each risk group. Funnel plots show outcomes in the context of precision, demonstrating how the institution performs compared to control limits (in this case, the 99% prediction interval around the calculated mean) [33].

The data structure for the analysis was hierarchical, with births (*n* = 1,475,457) at level 1, and hospitals (*n* = 1,373) at level 2 [34]. We used multilevel logistic regression models to quantify how much of the variation in cesarean section risk was attributable to hospitals. First we fit null models to describe the overall variation in cesarean section risk across hospitals for all births, and for births to women without prior cesarean sections. If the distribution of individuals with more medical complications caused hospital differences in the likelihood of cesarean section, we would expect to see less hospital-level variability in cesarean section use among the lower risk population than among the overall population, as measured by nonoverlapping credible intervals around the hospital variance estimate for these two groups. We then extended the null models to include maternal age, race/ethnicity, insurance status, and individual clinical diagnoses at level 1, and hospital bed size and location/teaching status and state at level 2. If individual clinical diagnoses—that is, medical conditions meeting diagnostic criteria—are driving hospital differences in the likelihood that an individual woman has a cesarean section, we would expect that accounting for clinical diagnoses and for hospital variables associated with greater resources or higher risk patients would reduce any hospital-level variability observed under null models. A significant reduction in the hospital variance (as indicated by nonoverlapping credible intervals after covariate adjustment) would suggest that hospital differences largely reflect the clustering of demographic and/or medical conditions of individuals by hospital. We tested a range of specifications for individual clinical diagnoses, and results were robust to these sensitivity analyses. Missing data were less than 5% for all variables except race/ethnicity (13%) and were handled using conventional methods in multilevel models. Data management tasks were conducted using SAS version 9.2. We used Markov chain Monte Carlo methods to fit Bayesian analytic models, where distributions for the model parameters were first estimated with predictive quasi-likelihood approximation with a second-order Taylor linearization procedure as implemented in MLwiN version 2.1 [35]. Bayesian models used a Metropolis-Hastings sampling algorithm, with the first 500 iterations dropped as burn in, and a chain of 5,000 iterations.

## Results

Table 1 presents descriptive information about the births (*n* = 1,475,457) and hospitals (*n* = 1,373) included in this analysis. The average hospital prevalence of cesarean section was 33.0% (95% confidence interval [CI] 32.9% to 33.1%) among all births, and the mean prevalence of primary cesarean section was 22.0% (95% CI 22.0% to 22.1%), defined among women with no prior cesarean sections (Table 1). Average risk of cesarean section and ranges were similar across hospitals of different sizes and location/teaching status for both groups of women. However, individual risk for cesarean delivery (among all women and among those with a prior cesarean section) varied by maternal age, race/ethnicity, and insurance status, and medical diagnoses related to pregnancy and delivery (Table 2).

**Table 1:** Characteristics of births (*n* = 1,475,457) and hospitals (*n* = 1,373) in the study population and among women with no prior cesareans, by hospital characteristics.

Characteristic	All Hospitals	Hospital Location/Teaching Status			Hospital Size		
		Rural and Not Teaching	Urban and Not Teaching	Urban and Teaching	Small	Medium	Large
**All births**							
Number of births	1,475,457	170,322	613,459	662,158	149,717	369,940	926,282
Number of hospitals	1,373	526	574	255	523	353	483
Number of states	46	44	41	39	46	45	44
Average cesarean rate for births	33.0%	31.7%	33.5%	32.9%	31.3%	32.7%	33.4%
Cesarean rate 95% confidence interval	32.9%, 33.1%	31.4%, 31.9%	33.4%, 33.6%	32.8%, 33.0%	31.1%, 31.5%	32.5%, 32.8%	33.3%, 33.5%
**Births to women with no prior cesareans**							
Number of births	1,241,255	144,470	515,565	556,501	126,985	311,867	777,684
Number of hospitals	1,372	526	573	255	522	353	483
Number of states	46	44	41	39	46	45	44
Average cesarean rate for births	22.0%	20.3%	22.1%	22.5%	20.2%	21.8%	22.5%
Cesarean rate 95% confidence interval	22.0%, 22.1%	20.1%, 20.5%	22.0%, 22.2%	22.4%, 22.6%	19.9%, 20.4%	21.6%, 21.9%	22.4%, 22.6%

29,518 births (2%) are missing data on hospital characteristics.

**Table 2:** US 2009–2010 births to all women and women with no prior cesarean: sample size, percentage frequency distribution, and percentage of women with cesarean deliveries and 95% confidence intervals by covariate.

Category	Characteristic or Diagnosis	All Births				Births to Women with No Prior Cesareans			
		*n*	Percent	Percent Cesarean Delivery	95% Confidence Interval	*n*	Percent	Percent Cesarean Delivery	95% Confidence Interval
**Patient diagnoses**	**Diabetes in pregnancy**								
	No	1,381,205	93.6%	31.9	31.9, 32.0	1,169,701	94.2%	21.3	21.2, 21.4
	Yes	94,252	6.4%	48.7	48.4, 49.1	71,554	5.8%	34.5	34.1, 34.8
	**Hypertension in pregnancy**								
	No	1,340,999	90.9%	31.3	31.3, 31.4	1,127,639	90.8%	20.1	20.0, 20.2
	Yes	134,458	9.1%	49.6	49.3, 49.9	113,616	9.2%	41.4	41.1, 41.7
	**Hemorrhage during pregnancy or placental complications**								
	No	1,449,237	98.2%	32.4	32.4, 32.5	1,219,857	98.3%	21.4	21.3, 21.5
	Yes	26,220	1.8%	64.3	63.7, 64.9	21,398	1.7%	57.9	57.2, 58.6
	**Fetal distress**								
	No	1,326,241	89.9%	30.1	30.0, 30.2	1,097,638	88.4%	17.3	17.3, 17.4
	Yes	149,216	10.1%	58.8	58.5, 59.0	143,617	11.6%	58.2	57.9, 58.4
	**Fetal disproportion or obstruction of labor**								
	No	1,408,214	95.4%	31.7	31.6, 31.8	1,178,553	94.9%	20.1	20.0, 20.2
	Yes	67,243	4.6%	60.3	59.9, 60.7	62,702	5.1%	58.4	58.0, 58.8
**Socio-demographics**	**Race/ethnicity**								
	White	673,867	52.7%	33.3	33.2, 33.4	571,003	53.2%	22.7	22.6, 22.8
	Black	188,752	14.8%	35.3	35.1, 35.6	157,151	14.6%	24.4	24.2, 24.6
	Hispanic	276,694	21.6%	32.4	32.2, 32.6	227,331	21.2%	19.6	19.5, 19.8
	Asian	64,572	5.1%	32.3	32.0, 32.7	54,850	5.1%	22.3	22.0, 22.7
	Native American	11,784	0.9%	33.3	32.5, 34.2	9,763	0.9%	21.0	20.2, 21.8
	Other	62,517	4.9%	33.3	32.9, 33.7	52,665	4.9%	22.9	22.5, 23.2
	**Insurance status (primary payer)**								
	Private	588,822	47.2%	35.4	35.3, 35.5	492,287	47.0%	24.3	24.2, 24.4
	Medicaid	553,598	44.4%	31.7	31.6, 31.8	462,798	44.2%	20.1	19.9, 20.2
	Medicare	28,231	2.3%	33.9	33.4, 34.5	26,844	2.6%	31.1	30.5, 31.6
	Other payment	32,241	2.6%	31.5	31.0, 32.0	27,553	2.6%	21.4	20.9, 21.9
	Uninsured	44,172	3.5%	29.6	29.2, 30.1	37,239	3.6%	18.9	18.5, 19.3
	**Age Category**								
	<30 y	890,618	60.4%	29.3	29.2, 29.4	778,799	62.8%	20.4	20.3, 20.5
	30–34 y	336,995	22.9%	36.6	36.5, 36.8	267,551	21.6%	22.6	22.4, 22.7
	≥35 y	246,473	16.7%	41.5	41.3, 41.7	193,696	15.6%	28.0	27.8, 28.2
**Hospital characteristics**	**Hospital location/teaching status**								
	Rural	170,322	11.6%	31.7	31.4, 31.9	144,470	11.9%	20.3	20.1, 20.5
	Urban, not teaching	613,459	41.6%	33.5	33.4, 33.6	515,565	42.4%	22.1	22.0, 22.2
	Urban, teaching	662,158	44.9%	32.9	32.8, 33.0	556,501	45.7%	22.5	22.4, 22.6
	**Hospital bed size**								
	Small	149,717	10.2%	31.3	31.1, 31.5	126,985	10.4%	20.2	19.9, 20.4
	Medium	369,940	25.1%	32.7	32.5, 32.8	311,867	25.6%	21.8	21.6, 21.9
	Large	926,282	62.8%	33.4	33.3, 33.5	777,684	63.9%	22.5	22.4, 22.6

Table 2 shows the sample size, percentage frequency distribution, cesarean section prevalence among women with each condition or characteristic, and 95% CIs for all study covariates. The demographic characteristics and clinical diagnoses, including race/ethnicity, age, and rates of hypertension and diabetes, were comparable to national estimates based on birth certificate data. Primary cesarean section prevalence was generally higher for women with diabetes in pregnancy (34.5% [95% CI 34.1% to 34.8%]), hypertension in pregnancy (41.4% [95% CI 41.1% to 41.7%]), hemorrhage during pregnancy or placental complications (57.9% [95% CI 57.2% to 58.6%]), fetal distress (58.2% [95% CI 57.9% to 58.4%]), fetopelvic disproportion or obstruction of labor (58.4% [95% CI 58.0% to 58.8%]), or maternal age ≥35 y (28.0% [95% CI 27.8% to 28.2%]). In addition, cesarean section prevalence (overall and primary) was higher among black women and women delivering at larger volume hospitals and hospitals in urban areas.

**Figure 1:**
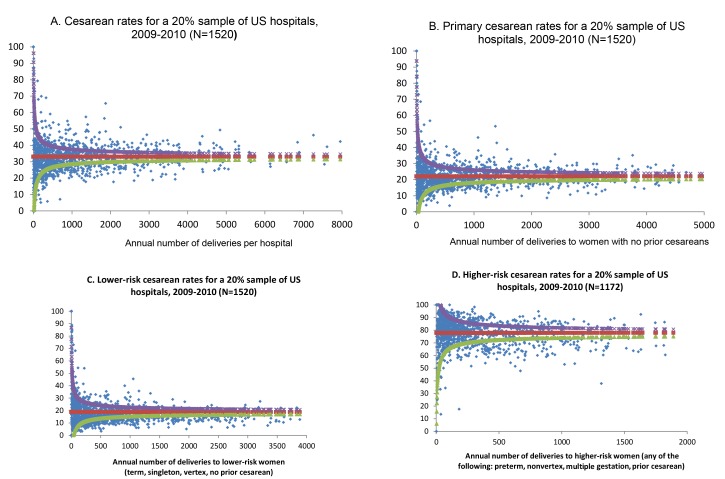
Funnel plots of hospital cesarean rates, overall and for subgroups of women. Funnel plots show how each individual institution (blue dot) performs compared to the mean (red) and control limits (the 99% prediction interval around the calculated mean). The upper control limit is shown as purple and the lower control limit is shown as green. Cesarean rates for (A) all women, (B) women with no prior cesarean, (C) lower risk women, and (D) higher risk women.

The distribution of cesarean section risk across the range of hospital delivery volumes indicates greater variability among hospitals with fewer deliveries, but similar overdispersion across the delivery volume spectrum, indicating that variability in cesarean section use exists for institutions of all sizes (Figure 1A). Based on chance, it would be expected that approximately 70 hospitals would fall outside the control limits, but instead 541 hospitals fell outside these limits. Figure 1B–1D shows funnel plots of hospital cesarean section rates for increasingly narrow groups of women, based on maternal risk status. Variability indicated by overdispersion outside of the control limits is present, even for more narrowly defined groups of women such as those with term, singleton, vertex pregnancies and no prior cesarean section, for whom cesarean section is less common, as well as for those with pregnancies at higher risk of cesarean section. There are several data points that are notable for their distance from the control limits; Figure 1B shows three hospitals with between 1,000 and 1,500 births per year to women with no prior cesarean sections that have a prevalence of cesarean sections among these women of between 40% and 55%.

Table 3 presents hospital variance and 95% credible intervals for null analyses and analyses fully adjusted for the covariates listed in Table 2, as well as state fixed effects, from a model of births nested in hospitals. In a model without adjustment for diagnosis of maternal hypertension, diabetes, hemorrhage or placental complications, fetal distress, and fetal disproportion or obstructed labor; maternal age, race/ethnicity, and insurance status; and hospital bed size and location/teaching status (null model), the hospital-level variance in the likelihood of an individual having a cesarean delivery, expressed on the logistic scale, was 0.13, and the 95% credible interval around this estimate excluded zero (95% credible interval 0.11 to 0.14) (Table 3). Expressed as a percentage, the risk of cesarean delivery varied between 19% and 48% across hospitals (range, 30 percentage points). After adjusting for individual diagnoses and the socio-demographic and hospital factors shown in Table 2, hospital-level variation did not decrease (0.14 [95% credible interval 0.12 to 0.15]).

**Table 3:** Hospital variance and 95% credible interval for null analyses and analyses fully adjusted for covariates listed in Table 2, from a multilevel model of births nested in hospitals.

Multilevel Regression Models	Hospital Variance (95% Credible Interval)
All births, null model	0.13 (0.11, 0.14)
All births, fully adjusted model	0.14 (0.12, 0.15)
All births to women with no prior cesarean, null model	0.14 (0.12, 0.15)
All births to women with no prior cesarean, fully adjusted model	0.16 (0.14, 0.18)
All births to lower risk women, null model	0.20 (0.18, 0.21)
All births to lower risk women, fully adjusted model	0.26 (0.23, 0.29)
All births to higher risk women, null model	0.30 (0.28, 0.34)
All births to higher risk women, fully adjusted model	0.25 (0.21, 0.28)

Among women with no prior cesarean section, hospital variability was similar (0.14 [95% credible interval 0.12 to 0.15]). Expressed as a percentage, the likelihood of an individual having a cesarean delivery varied between 11% and 36% across hospitals (range, 24 percentage points). Hospital-level variation in primary cesarean sections also did not decrease after adjusting for diagnosis of maternal hypertension, diabetes, hemorrhage or placental complications, fetal distress, and fetal disproportion or obstructed labor; maternal age, race/ethnicity, and insurance status; hospital bed size and location/teaching status; and state (0.16 [95% credible interval 0.14 to 0.18]).

We also examined two other subgroups of women, by risk level. Cesarean section risk among lower risk women (with term, singleton, vertex pregnancies and no prior cesarean sections) varied across hospitals (0.20 [95% credible interval 0.18 to 0.21]). Expressed as a percentage, the likelihood of a lower risk woman undergoing cesarean delivery varied between 8% and 32% across hospitals (range, 25 percentage points).Variance did not decrease after adjustment for maternal and hospital factors (0.26 [95% credible interval 0.23 to 0.29]).

Among the higher risk subgroup, there was greater variance in individual risk of cesarean section by hospital, but covariate adjustment resulted in a decrease in the point estimate of variance (however, this decrease was not statistically significant at *p*<0.05). Hospital-level variance in the likelihood of cesarean delivery among higher risk women was 0.30 (95% credible interval 0.28 to 0.34) before adjustment and 0.25 (95% credible interval 0.21 to 0.28) after controlling for maternal diagnoses and hospital factors. Expressed as a percentage, the likelihood of a higher risk woman having a cesarean delivery varied between 56% and 92% across hospitals (range, 35 percentage points). Parameter estimates for individual- and hospital-level covariates from the adjusted models are shown in Table 4 (overall cesarean section and primary cesarean section) and Table 5 (lower risk and higher risk subgroups).

**Table 4:** Parameter estimates from multilevel models of the association between patient and hospital covariates with odds of cesarean delivery, overall and among women with no prior cesareans.

Characteristic	All Births		No Prior Cesarean	
	OR	95% Credible Interval	OR	95% Credible Interval
Fetal distress	3.56***	3.51, 3.60	7.82***	7.71, 7.92
Gestational diabetes	1.84***	1.82, 1.87	1.79***	1.75, 1.82
Gestational hypertension	2.05***	2.02, 2.07	2.92***	2.88, 2.97
Hemorrhage during pregnancy or placenta problems	4.38***	4.26, 4.51	7.46***	7.23, 7.69
Fetopelvic disproportion or obstruction	3.06***	3.00, 3.12	5.61***	5.50, 5.72
Patient age	1.03***	1.03, 1.03	1.01***	1.01, 1.02
Black (white = reference)	1.14***	1.12, 1.15	1.08***	1.06, 1.10
Hispanic (white = reference)	0.96***	0.95, 0.97	0.81***	0.79, 0.82
Asian (white = reference)	0.86***	0.84, 0.87	0.85***	0.83, 0.88
Native (white = reference)	1.06*	1.01, 1.12	0.99	0.92, 1.05
Other race (white = reference)	0.97**	0.95, 0.99	0.97*	0.95, 1.00
Medicaid (private insurance = reference)	0.93***	0.92, 0.94	0.84***	0.82, 0.85
Medicare (private insurance = reference)	0.46***	0.44, 0.47	1.41***	1.36, 1.47
Other insurance (private insurance = reference)	0.93***	0.90, 0.95	0.94***	0.91, 0.97
Uninsured (private insurance = reference)	0.78***	0.76, 0.80	0.76***	0.74, 0.79
Urban, not teaching hospital (rural = reference)	1.01	0.96, 1.07	1.18***	1.12, 1.25
Urban, teaching hospital (rural = reference)	0.97	0.92, 1.03	1.18***	1.13, 1.24
Medium hospital size (small = reference)	1.08**	1.02, 1.14	1.07**	1.02, 1.13
Large hospital size (small = reference)	1.11***	1.07, 1.15	1.11***	1.06, 1.16

Models also control for state fixed effects. Bayesian one-tailed *p*-values based on posterior distributions.

*p<0.05,

**p<0.01,

***p<0.001.

OR, odds ratio.

**Table 5:** Parameter estimates from multilevel models of the association between patient and hospital covariates with odds of cesarean delivery for risk-based subgroups of women.

Characteristic	Lower Risk Births		Higher Risk Births	
	OR	95% Credible Interval	OR	95% Credible Interval
Fetal distress	13.64***	13.42, 13.86	0.74***	0.72, 0.77
Gestational diabetes	1.92***	1.87, 1.97	1.31***	1.27, 1.35
Gestational hypertension	2.84***	2.78, 2.90	1.27***	1.24, 1.30
Hemorrhage during pregnancy or placenta problems	10.63***	10.20, 11.08	1.01	0.96, 1.06
Fetopelvic disproportion or obstruction	7.65***	7.47, 7.83	2.32***	2.19, 2.46
Patient age	1.02***	1.01, 1.02	1.06***	1.06, 1.06
Black (white = reference)	1.26***	1.23, 1.28	0.90***	0.88, 0.93
Hispanic (white = reference)	0.82***	0.80, 0.83	1	0.98, 1.03
Asian (white = reference)	0.84***	0.81, 0.87	0.81***	0.77, 0.84
Native (white = reference)	1.04	0.96, 1.13	0.99	0.90, 1.09
Other race (white = reference)	0.99	0.96, 1.02	0.86***	0.82, 0.90
Medicaid (private insurance = reference)	0.87***	0.86, 0.88	0.98*	0.96, 1.00
Medicare (private insurance = reference)	2.29***	2.19, 2.39	0.92	0.83, 1.02
Other insurance (private insurance = reference)	0.96*	0.92, 1.00	0.92**	0.87, 0.98
Uninsured (private insurance = reference)	0.81***	0.77, 0.84	0.78***	0.74, 0.82
Urban, not teaching hospital (rural = reference)	1.21***	1.12, 1.31	0.91	0.82, 1.00
Urban, teaching hospital (rural = reference)	1.13**	1.03, 1.24	0.64***	0.57, 0.71
Medium hospital size (small = reference)	1	0.91, 1.10	0.93	0.85, 1.02
Large hospital size (small = reference)	1.03	0.95, 1.11	0.85***	0.79, 0.92

Models also control for state fixed effects. Bayesian one-tailed *p*-values based on posterior distributions.

*p<0.05,

**p<0.01,

***p<0.001.

OR, odds ratio.

**Figure 2:**
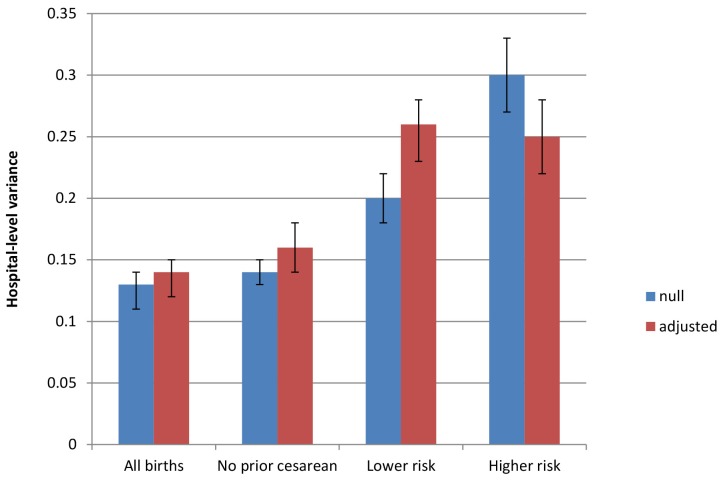
Between-hospital variation in cesarean deliveries overall and for subgroups of women, null and fully adjusted models. Populations include all births, all births to women with no prior cesarean delivery, all births to lower risk women (those with term, singleton, vertex pregnancies and no prior cesarean delivery), and all births to higher risk women (those with a preterm, multiple gestation, or nonvertex pregnancy or prior cesarean delivery). Factors included in adjusted models are diagnosis of maternal hypertension, diabetes, hemorrhage or placental complications, fetal distress, and fetal disproportion or obstructed labor; maternal age, race/ethnicity, and insurance status; hospital bed size and location/teaching status; and state-level fixed effects.

Figure 2 presents the between-hospital variation in the likelihood of having a cesarean delivery using both null and fully adjusted models for four groups (overall, low risk, primary, and high risk). Across all groups, adjustment for diagnosis of maternal hypertension, diabetes, hemorrhage or placental complications, fetal distress, and fetal disproportion or obstructed labor; maternal age, race/ethnicity, and insurance status; hospital bed size and location/teaching status; and state did not reduce hospital variation in the likelihood of a woman having a cesarean delivery.

## Discussion

Using data on all births that occurred in 2009 and 2010 in a nationally representative 20% sample of US hospitals, we find that variation in individual risk of cesarean section across hospitals is not explained by differences in maternal clinical diagnoses. Data on other aspects of individual clinical complexity (including parity and gestational age) as well as hospital factors (such as guidelines, protocols, and norms) is needed to enhance understanding of the drivers of variation in the individual likelihood of cesarean section across US hospitals.

### Comparison with Other Studies

The findings from this analysis are consistent with studies in Arizona, in Massachusetts, and among births in US military hospitals [20],[36],[37]; however, this analysis is the first to our knowledge to use a nationally representative US sample. These results provide an interesting comparison and counterpoint to research published in settings outside the US, where individual clinical characteristics accounted for a sizable portion of variability in the likelihood of cesarean section [21],[22],[38]. For example, a recent UK-based analysis indicated that approximately 1/3 of variability in cesarean section rates across National Health Service trust hospitals was attributable to patient case mix [22]. Parity was a strong independent predictor of cesarean section in this study, so the differences between the US- and UK-based findings may be due, in part, to differences in available data elements for analyses of this type. Whereas parity and gestational age are routinely available in population-based data sources in the UK [21],[22],[32], such data in the US are limited to birth certificates, which do not contain sufficient detail to calculate likelihood of cesarean section by hospital. The different findings in the UK versus the US also highlight some of the distinctions in maternity care management between the British and American health care systems, such as payment structures, out-of-pocket costs, and the role of midwife-led care, and contain valuable lessons for international application of these results.

The US is an outlier among high-income countries, with higher rates of health care spending but comparatively worse maternal and infant health outcomes [38],[39]. Coupled with our findings, this pattern implies potential inefficiencies that could be rectified to improve the quality of care in US hospitals. Contrary to findings in settings outside the US [21],[22], we found that the between-hospital variability did *not* decrease after accounting for maternal diagnoses and characteristics, suggesting that differences in the use of cesarean sections across hospitals are not diminished by comparing women with similar clinical conditions and basic socio-demographics. Maternal request for cesarean delivery may vary across hospital populations, but available data suggest that such requests constitute a very small percentage of all cesarean deliveries and do not likely drive the wide variations we detected [40]. In addition, our results indicate that between-hospital variability in risk of cesarean section remains substantial among women with no prior cesarean deliveries. These results therefore suggest that efforts to reduce unwarranted cesarean section variations—especially variations in use of primary cesarean sections—could gain traction through better data collection and reporting as well as a focus on hospital-level factors in order to inform implementation of current professional recommendations [41],[42].

These results add urgency to the need for more comprehensive data and evidence to inform ongoing clinical and policy efforts to reduce unnecessary cesarean sections and support consistent, high-quality maternity care worldwide, and in US hospitals in particular [13]–[15],[43]–[46]. Variability in procedure use reflects a potential lack of conformity to care standards and can indicate either overuse or underuse of services. Based on current rates, professional guidelines, and national public health goals, variation in the use of cesarean sections seems to be predominantly a problem of overuse [13],[41],[47], and one that requires remedies that include adoption of obstetric care guidelines and protocols [17],[41],[48].

However, the variability in prevalence of cesarean delivery among a higher risk subgroup of women also indicates that underuse is a potential issue that should not be overlooked in efforts to improve the appropriate use of cesarean sections. The comparatively higher inter-hospital variability estimates in this population imply a need for more research into the factors that might be driving hospital variability.

The major factors that may contribute to variability in obstetric practices include limitations in the clinical knowledge base and gaps in translation of evidence into changes in practice, as well as the roles of payers and medical liability concerns [49]–[51]. Addressing the gaps in knowledge and translation will require more comprehensive databases that include information on prenatal care, pregnancy characteristics, nonnondiagnostic clinical characteristics (such as parity and gestational age), hospital policies, and clinical care teams (including nurses and midwives as well as physicians). Our results also highlight the need for adherence to guidelines for use of cesarean delivery and for improving patient–provider communication and decision-making around childbirth care [16],[52],[53].

The results further suggest that efforts to implement guidelines should consider other aspects of care management, including, for example, hospital culture, practice patterns, management and administration, training needs, and organizational change.

In addition to efforts to routinely collect more comprehensive data to better understand variability in use of cesarean sections, hospitals, health care systems, hospital networks, and hospital associations can consider and assess adoption of available tools including guidelines, care “bundles,” and clinical protocols such as those the National Institute for Health and Care Excellence, Institute for Healthcare Improvement, Premier Perinatal Safety Initiative, and others have adopted [54]–[56]. The growing field of improved medical decision-making also emphasizes the importance of setting expectations, open communication, creating continuity across changes in staffing shifts and clinical conditions, providing informed consent, and a developing role for public reporting [19],[46]. Many other systems- or clinician-based factors may influence use of cesarean section in US hospitals [57], including liability and insurance factors [36],[50],[51],[58],[59], the presence and type of a hospital's clinical training program [36],[37],[60], the role of midwifery [61],[62], the presence of labor support or birth doulas [63],[64], individual clinician approaches to labor and delivery management [60],[65],[66], and practices related to admission and labor management [44],[67]. It will be important for future research to examine these factors, including whether clinician styles drive hospital effects (i.e., clinicians “sorting” themselves into hospitals) and how hospital policies influence practice patterns (e.g., consistent with “learning health care systems” and continuous quality improvement) [68],[69].

### Strengths and Limitations

Several limitations of our analysis merit discussion. As previously mentioned, there are crucial elements that are not included in the hospital administrative data used for this analysis. Although the NIS data are reliably coded and have been successfully used in prior analyses of obstetric care outcomes [29],[70], we were unable to identify nulliparous women in this dataset, nor was information on gestational age available. Having such information would have enhanced our ability to adjust for risk and would have enabled calculation of the nulliparous, term, singleton, vertex (NSTV) cesarean section rate or classification by the Robson cesarean classification system, which are commonly used metrics [32]. Parity is a strong predictor of cesarean section risk, as nulliparous women have higher risk of cesarean section after labor begins, multiparous women with a prior cesarean section have higher likelihood of prelabor or planned cesarean sections, and multiparous women without a prior cesarean section are more likely to have a spontaneous vaginal birth [21]. The relationship between gestational age and cesarean section is nonlinear. Likelihood of cesarean section is higher for preterm (<37 wk gestation) and for post-term (>41 wk) births. In this analysis, we accounted for prior cesarean section and preterm birth, but data on other aspects of parity and gestational age were not included in this administrative data source. The lack of information on parity and gestational age is a major limitation of hospital discharge data, and future efforts toward adoption of health information technology and interoperability of electronic health records should focus on facilitating access to these important data elements for hospitals that aim to improve maternity care quality.

Observed variability in cesarean use across hospitals could be overestimated because of limitations of the data. For instance, the case mix of women's parity and gestational age could have varied between hospitals, and thus any inference on “true” hospital variability could be confounded by unmeasured factors at the individual level. While plausible, this potential threat to the validity of our findings needs to be viewed in the broader context of our analysis. To invalidate our key findings, the unmeasured risk factors have to both be a prior common cause to the likelihood of an individual having a cesarean section (which is the case for parity and gestational age [20],[36],[42]) *and* be clustered by hospitals in the same manner as the clustering of cesarean section. As we show in Table S1, while measured clinical diagnoses (e.g., hypertension, diabetes, placental complications, fetal distress, and fetal disproportion) are associated with substantially increased odds of cesarean section for individual women, they do not explain any of the between-hospital variability in cesarean section rates, either overall or for women with no prior cesareans. For the measured risk factors in our analysis, these factors were not a confounder of variability between hospitals in use of cesarean. Nonetheless, future research should collect and include data to assess the contribution of other risk factors (such as gestational age and parity) that we could not measure to explain hospital variability.

Furthermore, discharge data do not contain clinical details on reasons for cesarean delivery or hospital-level information on obstetric care guidelines and policies, which constrains our ability to assess the appropriateness of care or many possible administrative or clinical explanations for variations in cesarean section rates across hospitals. There are no nationally representative datasets in the US that contain a greater level of detail on childbirth-related health care services than the data we used in this study; however, future studies using linked datasets offer promise for more comprehensive analyses on this topic. Our analyses do not include clinician-level information because of data limitations, so differences due to the specialty training or discipline of the attending clinicians within a hospital (e.g., midwifery, family medicine, maternal–fetal medicine) cannot be measured. In spite of these limitations, our analysis offers important new information on variation in the use of cesarean sections in US hospitals by utilizing a nationally representative administrative data source.

## Conclusions

There is substantial variation in use of cesarean section across hospitals in the US. Hospital variability in the likelihood of a woman having a cesarean section is not decreased by accounting for diagnosis of maternal hypertension, diabetes, hemorrhage or placental complications, fetal distress, fetal disproportion or obstructed labor, maternal age, race/ethnicity, insurance status, or hospital factors. The data analyzed here, while nationally representative, did not contain information on parity or gestational age; future research must examine these important factors. The current findings highlight the need for more comprehensive data and examination of other factors—such as hospital policies, practices, and culture—in determining cesarean section use.

## Supporting Information

Table S1 Predictors of individual cesarean risk and predictors of hospital variance in likelihood of cesarean section, for all women and for women with no prior cesarean section.
(DOCX)

Checklist S1 Strobe statement.
(DOC)

## Abbreviations

AHRQ: Agency for Healthcare Research and Quality
CCS: Clinical Classifications Software
CI: confidence interval
HCUP: Healthcare Cost and Utilization Project
ICD-9: International Classification of Diseases, 9th revision
NIS: Nationwide Inpatient Sample
