# Supplementary material for: Maternal Clinical Diagnoses and Hospital Variation in the Risk of Cesarean Delivery: Analyses of a National US Hospital Discharge Database
Source: PLoS Med. 2014 Oct 21;11(10):e1001745. doi: 10.1371/journal.pmed.1001745 (PMC4205118; doi:10.1371/journal.pmed.1001745)
Supplement: Table S1 — Predictors of individual cesarean risk and predictors of hospital variance in likelihood of cesarean section, for all women and for women with no prior cesarean section. (DOCX) [file pmed.1001745.s001.docx]

| **Table S1: Predictors of individual cesarean risk and predictors of hospital variance in likelihood of cesarean section, for all women and for women with no prior cesarean section.** | | | | | | | | |
| --- | --- | --- | --- | --- | --- | --- | --- | --- |
|  | **All women** | | | | **Women with no prior cesarean section** | | | |
|  | *Individual cesarean risk* | | *Between hospital variability in cesarean rates* | | *Individual cesarean risk* | | *Between hospital variability in cesarean rates* | |
| **Patient clinical diagnosis** | OR | 95% CI | hospital variance | 95% CI | OR | 95% CI | hospital variance | 95% CI |
| Null | - | - | 0.12 | 0.11,0.13 | - | - | 0.14 | 0.12,0.15 |
| Hypertension | 2.17 | 2.14,2.19 | 0.12 | 0.11,0.13 | 2.82 | 2.78,2.85 | 0.14 | 0.13,0.16 |
| Diabetes | 2.03 | 2.01,2.06 | 0.12 | 0.11,0.13 | 1.94 | 1.91,1.97 | 0.14 | 0.13,0.15 |
| Placenta disorders | 3.73 | 3.63,3.82 | 0.12 | 0.11,0.13 | 5.00 | 4.87,5.14 | 0.14 | 0.13,0.15 |
| Fetal distress | 3.39 | 3.35,3.43 | 0.13 | 0.12,0.14 | 6.85 | 6.77,6.93 | 0.20 | 0.18,0.22 |
| Fetal disproportion/ obstruction | 3.26 | 3.21,3.32 | 0.12 | 0.11,0.13 | 5.59 | 5.50,5.69 | 0.14 | 0.13,0.16 |
| Notes: Individual cesarean risk models use bivariate logistic regression, and between-hospital variability in cesarean rates is estimated using iterative generalized least squares estimates. For all results shown, p<0.001 | | | | | | | | |
